# Effect of RG (Coptis root and ginseng) formula in patients with type 2 diabetes mellitus: a study protocol for a randomized controlled and double-blinding trial

**DOI:** 10.1186/s13063-022-06229-5

**Published:** 2022-04-14

**Authors:** Li Jiang, Qiang Fu, Shidong Wang, Yu Chen, Jiayue Li, Yonghua Xiao, Ruixi Sun, Esther Aijia Shen, Junheng Wang, Yaofu Zhang, Zhuang Li, Jiangteng Liu, Xiaozhe Fu, Yuanyuan Liu, Yu Zhao, Guanxun Su, Weijun Huang, Jinxi Zhao

**Affiliations:** 1 Graduate School, Beijing University of Chinese Medicine, Beijing, China; 2 Section II of Endocrinology & Nephropathy department of Dongzhimen Hospital affiliated to Beijing University of Chinese Medicine, No.5 Haiyuncang Road, Dongcheng District, Beijing, China; 3 Key Laboratory of Chinese Internal Medicine of Ministry of Education and Beijing, Dongzhimen Hospital affiliated to Beijing University of Chinese Medicine, Beijing, China

**Keywords:** Protocol, Type 2 diabetes mellitus, TCM, Gut microbiota, Randomized controlled trial

## Abstract

**Background:**

Type 2 diabetes mellitus (T2DM) is a common metabolic disease with significant health, social, and economic consequences. Traditional Chinese medicine (TCM) could effectively regulate blood sugar and influence gut microbiota in T2DM patients. Preliminary studies showed that the Coptis root and ginseng (RG) formula could relieve insulin resistance and prevent the progression of diabetes in mice.

**Objectives:**

The purpose of this study is to explore the efficacy and safety of RG formula in the treatment of adult patients with T2DM, as well as observing its effects on gut microbiota.

**Methods and analysis:**

This trial is a randomized, double-blind, placebo-controlled study. A total of 60 participants will be randomized in a 1:1 ratio into an experiment group (RG formula) and a control group (placebo). Patients in both groups will be given diabetes education and basic blood glucose control. Glucose-lowering drugs with significant influence on gut microbiota will be avoided. This trial will last 25 weeks including 1-week run-in, 12-week intervention, and 12-week follow-up visit. The primary outcome is the change in the HbA1c. The secondary outcomes comprise the change in the fasting blood glucose (FBG), postprandial blood glucose (PBG), fasting insulin (FIL), fasting C-peptide(C-P), insulin resistance index (IRI), inflammatory factors, and species abundance of gut microbiota between the two groups. Safety of medication will also be evaluated. The correlation analysis will be explored between the glycemic indicators, inflammatory factors, and abundance of gut microbiota.

**Discussion:**

This study will provide the clinical evidence for the efficacy of RG formula in regulating blood sugar and influencing gut microbiota, which will be beneficial to form the integrated therapeutic regimen in T2DM with TCM.

**Trial registration:**

“Clinical Study on the Intervention of Coptis Root and Ginseng,” Chinese Clinical Trials Registry ChiCTR 2100042126. Registered on 14 January 2021

**Supplementary Information:**

The online version contains supplementary material available at 10.1186/s13063-022-06229-5.

## Background

Diabetes mellitus (DM), which is caused by absolute or relative insulin deficiency and reduced sensitivity of target cells to insulin [1], is a common metabolic disease with significant health, social, and economic consequences. Around 1 in 11 adults worldwide now have been diagnosed with DM, 90% of whom are type 2 diabetes mellitus (T2DM) [2]. According to the 2017 survey by China’s National Center for Disease Control and Endocrinology Branch of the Chinese Medical Association, the prevalence rate of T2DM in China was 12.8%. China has become the country with the largest number of diabetes patients worldwide [3]. Patients with T2DM have a twofold to tenfold higher risk of cardiovascular disease-related death than age-matched normoglycemic individuals and are at substantially higher risk of all-cause mortality, cardiovascular complications (such as coronary heart disease, heart failure, stroke, and peripheral arterial disease), and microvascular complications (including retinopathy, neuropathy, and nephropathy), which seriously threatens their quality of life and brings heavy burden to the society [4].

Gut microbiota is closely related to diabetes. A Chinese research team has clarified the differences in gut microbiota between diabetic patients and healthy people at the molecular level by using metagenomic association analysis [5]. A series of studies in Gordon’s lab showed that the gut microbiota in patients with T2DM was characterized by a decrease in the abundance of butyrate-producing bacteria, such as *Bacteroidetes*, and an increase in various opportunistic pathogens, such as *Firmicutes* [6, 7]. Larsen found that in diabetic patients *Firmicutes* including *Clostridium* significantly reduced, while *Proteobacteria* which was positively correlated with blood glucose concentration significantly increased [8]. From normal glucose tolerance to pre-diabetes and then to the occurrence of T2DM, the relative abundance of *Streptococcus* continued to decline, while the relative abundance of *Verrucosa* and *Proteobacteria* showed an opposite trend, indicating that dysregulation of gut microbiota was associated with the progression of T2DM [9]. Some scholars proposed that gut microbiota influenced the occurrence and development of T2DM mainly through mechanisms such as the effect of intestinal energy acquisition [10–12], regulation of fat storage, influence of adipokines [13–15], and regulation of inflammation induced by metabolic endotoxemia [16].

Traditional Chinese medicine (TCM) could treat T2DM by regulating the disturbance of gut microbiota. A meta-analysis on TCM improvement of gut microbiota in the treatment of T2DM showed that TCM generally reduced fasting blood glucose and postprandial blood glucose and improved insulin resistance by means of enriching the relative abundance of bacteria in the genera *Bacteroides* [17]. To a specific degree, different TCM interventions could play a complex role in the various bacteria. On the one hand, the traditional formula such as GGQL and AMC formula has been proved to remodel the structure of gut microbiota. The GGQL formula, which consists of Radix Puerariae, *Scutellaria baicalensis*, Coptis root, and Radix liquiritiae, could enrich the abundance of intestinal beneficial bacteria like *Clostridium leptum* and inhibit the growth of intestinal pathogenic bacteria like *Fusobacterium*, thus improving the permeability of intestinal mucosa and treating T2DM [18]. The AMC formula, whose components are Rhizoma Anemarrhenae, Momordica charantia, Coptis root, Aloe vera, and red yeast rice, may ameliorate T2DM with hyperlipidemia by enriching beneficial bacteria, such as *Blautia* and *Faecalibacterium* spp. [19]. On the other hand, TCM extracts like ginseng polysaccharides and berberine also had a good effect in modulating gut microbiota structure as a potential mechanism for the improvement of T2DM. Studies showed that berberine could selectively inhibit the growth of *Blautia*, *Allobaculum* [20], *Ruminococcus gnavus*, *Ruminococcus schinkii*, *Lactobacillus acidophilus*, *Lactobacillus murinus*, and *Lactococcus lactis* [21], which may explain its effect on improving adipose infiltration and insulin resistance in high-fat diet-fed rats. Ginseng polysaccharides were confirmed to reverse the dysbiosis of intestinal flora in diabetic rats at the phyla level to approach the homeostasis mainly by increasing the relative abundance of *Firmicutes* and decreasing that of *Bacteroidetes* [22].

Previous studies found that the formula of Coptis root and ginseng (RG) could reduce postprandial blood glucose by inhibiting intestinal α-glucosidase in T2DM patients [23]. Our team also demonstrated that the RG formula could alleviate insulin resistance in diabetic rats through inhibiting the expressions of pro-inflammatory factors and pro-apoptotic factors in pancreatic islets, up-regulating adiponectin receptors, and reducing the level of serum-free fatty acids [24, 25]. Whether long-term intervention of RG formula will remodel the structure of gut microbiota, reduce inflammation, and improve insulin resistance is worthy to be explored. Based on the viewpoint of gut microbiota, this study aims to (1) assess the clinical efficacy of RG formula in adults with T2DM, (2) characterize the influence of RG formula on levels of inflammatory cytokines and structural changes of gut microbiota, and (3) analyze the relationship between changes in gut microbiota, inflammatory cytokines, and clinical assessments, in order to demonstrate the underlying mechanism of TCM treatment in T2DM patients.

## Methods

### Study design

**Fig. 1:**
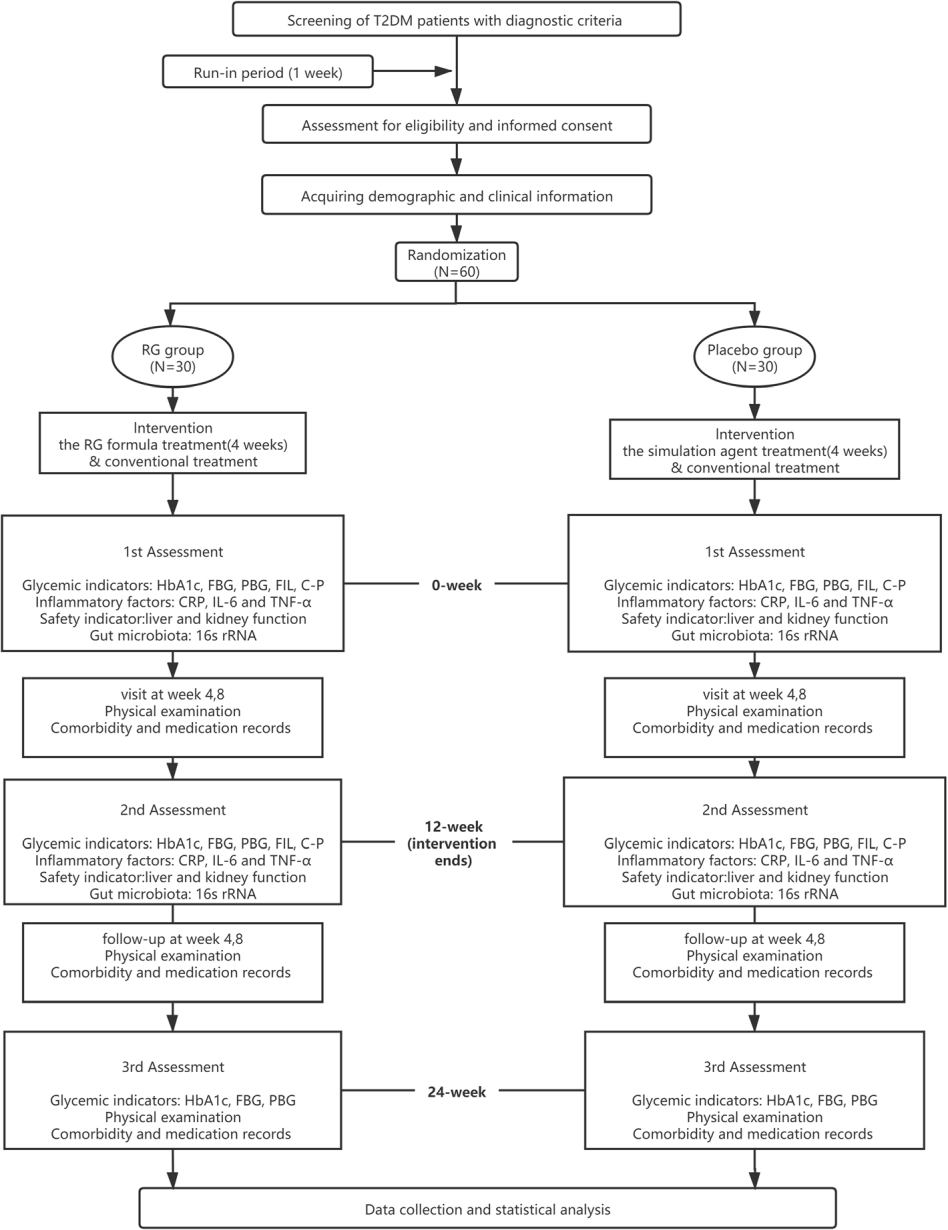
The trial flow diagram

This protocol is designed as a randomized and placebo-controlled trial. Participants and investigators are blinded. A total of 60 subjects will be recruited at the Section II of Endocrinology & Nephropathy department of Dongzhimen Hospital affiliated to Beijing University of Chinese Medicine. They will be randomized in a 1:1 ratio to an experiment group (RG formula) and a control group (placebo). Patients in both groups are given basic blood glucose control. Only the use of insulin and insulin secretion promoters (such as thiazolidinedione and sulfonylurea) can be allowed as basic control in both groups, as current research has shown no significant influence on gut microbiota in neither drugs. The trial flow diagram is illustrated in Fig. 1. The SPIRIT Checklist is shown in Supplemental file 1 [26].

### Participants

#### Diagnostic criteria

The diagnostic criteria for this trial will be set based on the American Diabetes Association (ADA) guidelines in 2018 [27]: (1) Patients with typical diabetes symptoms and random blood glucose ≥11.1 mmol/L or random venous plasma glucose ≥11.1 mmol/L. Typical symptoms of diabetes include polydipsia, polyphagia, polyuria, dysphoria, and weight loss without other triggers; (2) FBG or venous plasma glucose ≥ 7.0 mmol/L, which is defined as no calorie intake for at least 8 h; (3) PBG after OGTT test ≥ 11.1 mmol/L; and (4) HbA1c ≥ 6.5%.

#### Inclusion criteria

Patients who meet the following criteria will be considered for enrollment: (1) age 18–65; (2) meet the diabetes diagnostic criteria of ADA; (3) FBG is stable at less than 7 mmol/L and PBG is stable at less than 10 mmol/L; and (4) informed and consented. The process of obtaining informed consent shall comply with Good Clinical Practice (GCP).

#### Exclusion criteria

Patients are excluded if any of the exclusion criteria are met: (1) new-onset of T2DM, who should take metformin orally as recommended by guidelines ; (2) patients with cardiac insufficiency (B-type natriuretic peptide is greater than the upper limit of normal) or with impaired liver function (alanine transaminase and aspartate aminotransferase are twice greater than the upper limit of normal) or impaired kidney function (serum creatinine is greater than the upper limit of normal) or mental illness; (3) patients who had diabetic ketosis, diabetic ketoacidosis, or severe infection within 1 month before recruitment; (4) patients who are allergic to Coptis root or ginseng; (5) patients who receive antibiotics and weight-reducing medicine or have participated in other clinical trials within 1 month before recruitment; (6) patients who have inflammatory bowel diseases like ulcerative colitis and Crohn’s disease; and (7) pregnant or lactating women.

#### Withdrawal criteria

Participants who meet any of the following conditions will be removed from the study: (1) those who have poor medication adherence during the run-in period; (2) who can hardly not tolerate the side effect of experimental medicine; (3) who have severe cardiovascular and cerebrovascular complications or infections during the study observation; (4) who are unable to participate in the following visit in time within a week and refuse to be contacted after more than three telephone communication by researchers; and (5) who have intermittent use of probiotics and other Chinese patent medicine during the study.

### Study setting

The treatment will be conducted at Dongzhimen Hospital affiliated to Beijing University of Chinese Medicine. The study will enroll 60 individuals in total, with 30 individuals in each group. Patients who are included in the study are required to sign an informed consent form. The researchers will spend 10–15 min informing the patients of the benefits and possible risks according to the statement in the informed consent. The informed consent form is provided in Supplemental file 2. An overview of specific measurements and time points of data collection is provided in Table 1.

**Table 1:** Measurement items and point of data capture

Visit project		Screening period/baseline	Visit 1	Visit 2	Visit 3	Visit 4-6
Visiting time		–7 to 0 days	Medication week 4	Medication week 8	Medication week 12	Follow-up weeks 4, 8, and 12
Collect basic medical history						
Sign informed consent		√				
Fill in general information		√				
Medical history and treatment history		√				
Determine inclusion and exclusion criteria		√				
Physical examination		√	√	√	√	√
Comorbidity and medication records		√	√	√	√	√
Effectiveness observation						
Glycosylated hemoglobin		√			√	√
Fasting blood glucose		√			√	√
Postprandial blood glucose		√			√	√
Fasting insulin		√			√	
Fasting C-peptide		√			√	
Inflammatory factors		√			√	
Other work						
Random grouping		√				
Fecal specimen		√			√	√
Liver and kidney function		√			√	√
Adverse event		√	√	√	√	√

### Participant recruitment

The trial subjects will be recruited from the outpatient and the ward in the Section II of Endocrinology & Nephropathy department and the community around the Dongzhimen hospital. A multipronged approach is used to identify potential participants to reach the required sample size: ①Advertisement through posters and pamphlets (placed/distributed at various locations including these notice boards in hospitals, outpatient receptions, communities, parks, and health clinics), in which brief description of the subjects that are eligible and details of contact and necessary contributions as participants would be covered. ②An incentive of 100¥ would be offered to physicians or resident doctors for each patient referred by their practice who could be successfully recruited for the trial.③Door-to-door visits in surrounding communities that have the collaboration with Dongzhimen hospital at various times. The investigator will introduce the protocol as well as the benefits and risks to the enrolled participants and an informed consent form is mandatory before the study.

Patients recruited from other hospitals through advertisement will be required to undergo a week of observation, including the control of blood glucose, medication compliance, and degrees of resistance. Following the observation, subjects that meet the inclusion criteria can then be admitted into this clinical trial. The following basic personal information will be collected: sex, age, body mass index (BMI), educational background, occupation, marital status, past medical history, etc. Patients’ personal information is under the supervision of the Data Monitoring Committee (DMC), and this information is protected and will not be disclosed to any individual or organization unrelated to this study.

### Randomization and blinding

#### Sequence generation

This clinical research adopts a randomized, double-blind, parallel-group study method. Patients who meet the criterion of T2DM are randomly divided into the control group and the experimental group. Randomization is achieved by using the random number table method in Statistical Analysis System (SAS 9.2), which is operated by a statistician blinded to treatment and data collection.

#### Allocation concealment mechanism

The random number sequence generated by the system will correspond to the number of the sixty subjects one by one. These numbers are then arranged from small to large (in those with the same random number, the one which is picked out first will be arranged in the front). One to 30 will be designated as the experimental group while 31–60 are the control group. The documents of the random process and randomization list will be enclosed in an opaque envelope. Participants, researchers, and coordinators together with statisticians are not aware of the allocation of the patient and remain unaware until trial completion. The randomization of allocation and concealment of allocation are completed by two researchers who do not communicate with each other.

#### Implementation

The group manager will send an answer form to the investigator who is not involved in assessing the outcome of the study. This form will include a randomization number. The closed envelopes with printed randomization numbers on them are available. For every randomization number, the corresponding code of the randomization list will be found inside the envelopes. The investigator will open the envelope and will find the appropriate drugs to be conducted in this patient without giving the information about treatment allocation to the patient. Staff responsible for recruitment and following up is not allowed to receive information about the group allocation. The allocation sequence will be generated by the Key Laboratory of Chinese Internal Medicine of the Ministry of Education. Throughout the study, the randomization will be conducted by Section II of Endocrinology & Nephropathy department in order to keep the data management and the statistician blind against the study condition as long as the data bank is open. The randomization list remains with the group manager for the whole duration of the study. Thus, randomization will be conducted without any influence of the principal investigators, statistician, or physician.

#### Blinding

In this trial, we use the simulation agent made of dextrin and condiment as the placebo. Both RG formula and placebo are manufactured by Kang Taisheng Pharmaceutical Co., Ltd. with the same specification, appearance, and flavor. The independent statistician, along with the Ethics committee of the study center, is responsible to monitor and assess the blinding procedure as part of data monitoring. The blind code will be disclosed after completion of the statistical analysis.

### Intervention

Patients in both groups are given the same diabetes education and lifestyle intervention. After basic blood glucose control, the fasting blood glucose is stable at less than 7 mmol/L and postprandial blood glucose is stable at less than 10 mmol/L. In this process, metformin, GSDI (α-glucosidase inhibitor), DPP-4 (dipeptidyl peptidase 4) inhibitor, and GLP-1 (glucagon-like peptide-1) analogue are avoided. Insulin and insulin secretion promoters are allowed. On the basis of standard treatment, the experimental group is given a TCM granule packet. Each packet contains 11 g RG formula, which includes 10 g Coptis root and 1 g ginseng. Patients will take RG formula at least for 3 months, once daily, 11 g each time. The control group is given the same amount of Chinese medicine simulation agent which is taken by the same way. The simulation agent contains dextrin and condiment, which looks and tastes exactly like the RG formula. The course of intervention is 12 weeks, and medicine is given every 4 weeks. Serum and stool samples are collected at baseline and at week 12 for relevant indicators. During the trial, the subjects are forbidden to use other TCM drugs (including proprietary Chinese medicine, TCM injections, etc.). For patients that have other chronic and/or complicated diseases that require long-term control/lifetime medication that does not contradict with inclusion criteria, thus, they can be exempted from medicinal limitation; however, this information should be recorded in detail in the case report form (CRF). Follow-up observation is continued after 12 weeks of administration to determine the subsequent effects of the drug. Dongzhimen Hospital affiliated to Beijing University of Chinese Medicine has insurance to cover for non-negligent harm associated with the protocol. This will include cover for additional health care, compensation, or damages whether awarded voluntarily by the sponsor or by claims pursued through the courts. Should this study provide evidence of the effectiveness of RG formula in controlling blood glucose and relieving discomfort, it will be recommended for the patients to continue taking the medicine with a half or less dose as the dietary supplements. The detailed component of this formula will be informed to subjects and these two traditional herbs are common and available in most pharmacies as over the counter. If the subjects are prone to receive the conventional medicine, the consultation services on the online platform will be provided up to 3 months to help patients to develop a personalized glycemic schedule. This study has been approved by the Ethics Committee of Dongzhimen Hospital.

### Outcome measures

The physical condition of patients should be recorded at baseline, week 4, week 8, and week 12.

At the start of the trial, patient’s general information including name, gender, age, education, marriage condition, BMI, etc., will be collected. The complications of diabetes such as diabetic retinopathy, diabetic nephropathy, diabetic peripheral neuropathy, and diabetic peripheral vascular disease should be noted. Other diseases and their corresponding treatment, symptoms, tongue and pulse condition of TCM should also be collected. At week 4 and week 8, two follow-up visits are conducted to collect TCM-related indicators such as symptoms, tongue signs, pulse signs, and symptom diagnosis. At week 0 and week 12, fecal samples and blood samples are collected for quantitative determination of gut microbiota, serum biochemical indicators, and inflammatory indicators.

Stool sample measuring is based on the 16S rRNA sequencing technology of microbiome. It will detect qualified environmental microbial DNA extraction and make the PCR amplification for the general area in V3-V4 primer in DNA base sequence of the designated areas. After a large number of replicated increments, the result will be compared to the existing databank. When the sample passes the inspection, DNA data will be uploaded to Illumina Hiseq 2500 high-throughput sequencing platform for OTU annotation and relative abundance analysis, so as to calculate the species abundance of all samples at phylum, class, order, family, and genus levels. On the basis of these results, the clustering of species abundance, association analysis of common specific OTU, and inter-group differential species analysis will be completed.

Measures of blood samples are divided into biochemical index and inflammation index. As for biochemical index, FBG and PBG are measured by hexokinase method, HbA1c by glycosylated hemoglobin automatic analyzer using the ion-exchange HPLC method, insulin and C peptide by the chemiluminescence method, urea by the colorimetric method, creatinine values by the sarcosine oxidase method, and ALT and AST by the rate method. As for inflammatory indicators, IL-6, TNF-α, and CRP are determined by professional operators using relevant detection kits and enzyme-labeled analyzer (Infinite F50).

#### Primary outcomes

The primary outcome in this study is glycosylated hemoglobin (HbA1c), which means the comparison of HbA1c change between two groups after 12 weeks of intervention. HbA1c is a critical indicator for the diagnosis of T2DM, which can reflect average levels of glycemia during the preceding 3 months and is widely used for diabetic complication assessment [28]. It is considered the gold standard for predicting glycemia-associated risks for the microvascular and macrovascular complications of diabetes mellitus over 5–10 years [29]. Therefore, the value of HbA1c in the care of patients with T1DM and T2DM is unassailable [30]. Professional societies, public health organizations, regulatory agencies, patients, and clinicians have focused on HbA1c levels to gauge the quality of diabetes care, and over time, HbA1c level has supplanted other indicators of the quality of diabetes care, such as blood glucose levels and symptoms of hyperglycemia, despite being a surrogate rather than a direct marker of glycemic control [31]. In addition, careful regulation of blood glucose concentrations in patients with diabetes mellitus normalized HbA1c over 6 weeks [32], so it has also excellent reliability and validity and is sensitive enough for clinical and research practice.

#### Secondary outcomes

The secondary outcomes are mean changes in FBG, PBG, FIL, C-P, IRI, inflammatory factors, and species abundance of gut microbiota. FBG, PBG, FIL, and C-P will be measured to further assess the status of insulin resistance. PBG is obtained by rapid detection of fingertip blood. FBG, FIL, and C-P are obtained by venous blood sample which will be collected after an overnight by a trained medical officer/investigator and transported to a standard laboratory for analysis. Laboratory technicians are blinded to the study participants. The insulin resistance index of the homeostasis model is based on the formula developed by Matthews [33]. The inflammatory factors include interleukin-6 (IL-6), tumor necrosis factor α (TNF-α), and C-reactive protein (CRP). The species abundance of gut microbiota will be based on the second-generation high-throughput sequencing technology.

#### Safety assessment

Safety indicators including blood routine and urine routine and liver and kidney function are measured before and after the study. Adverse events, such as hypoglycemic reactions, constipation, and abdominal pain, are recorded in detail and their association with medication is determined by professional researchers. The trial can be terminated if necessary. During the study period, the adverse event record form is completed to record the duration, occurrence time, severity, measures, and outcome of the adverse event. The severity of the adverse events is divided into mild, moderate, and severe. Mild can be perceived as a tolerable adverse effect and does not require immediate medical attention; moderate is intolerable and requires special treatment; severe discomfort requires timely termination of the trial and immediate emergency treatment. Researchers will report it to the person in charge of the trial, the sponsoring unit, and the medical ethics committee in time, and even report it to the adverse reaction center according to the relevant regulations of the State Medical Products Administration. The ethics committee have the right to make the final decision if termination is needed.

In this study, Coptis root is known to have the effect of promoting blood circulation and dissipating blood stasis. Therefore, the risk of increased menstrual bleeding should be clearly informed when female subjects are enrolled. Aside from signing the informed consent, the female subjects are also instructed to take a bag of drugs 2–3 times during menstruation to reduce the peak blood drug concentration and the risk of heavy menstruation. Last but not least, if the amount of bleeding is large, an authorized clinician should complete the judgment of causality and exclude the menstrual abnormalities caused by other factors, such as the combined effect of conventional drugs, the progress of the subject’s primary disease, and other treatment measures. Should large menstruation is confirmed and recorded as adverse reactions, the reduction of dosage or drug withdrawal would be necessary. In addition, there is also the possibility for patients to experience diarrhea, abdominal pain, and secondary constipation. If diarrhea and abdominal pain occur, then the patients will be suggested to take the medicine within 30 min after the meal so as to reduce the direct stimulation of Coptis root in the gastrointestinal tract. If constipation appears, then patients are suggested to take the medicine with honey, as TCM herbal theory believes it has the effect of promoting bowel movement by moistening the large intestine.

### Sample size

Sixty participants (*n* = 60) will be recruited after the run-in period and allocated to the RG group (*n* = 30) and the placebo group (*n* = 30). The sample size was calculated considering the expected change of glycosylated hemoglobin (HbA1c) (primary outcome) of the participants by software PASS15.0. Previous studies have reported that formula including Coptis root and ginseng had statistically and clinically significant reduction of absolute HbA1c by about 0.72% [34, 35] and mean change in HbA1c by about 0.70% [36]. The sample size was calculated to have 80% power to detect a moderate 0.65-SD difference in HbA1c, with an alpha/*α* value of 0.05. Additional participants will be recruited until the required number is achieved (*n* = 30) per group to account for the estimated drop-out rate of 15%. Therefore, we take the sample size as 30 in both groups.

### Patient and public involvement

Patients in this trial will not be involved in the design, recruitment, or conduction of the study. After the measure of the samples, the patients will be informed of the results of indicator data in the form of report. The doctor in charge of the patient will be also informed of the results to optimize treatment and the rehabilitation scheme.

### Adherence to study medication

Strategies to improve adherence to intervention protocols include:

(1) Providing professional lifestyle guidance, 2 weeks of TCM treatment, and laboratory index containing liver function, kidney function, HbA1c, fasting blood glucose, postprandial blood glucose, and gut microbiota for free. The outcomes of HbA1c, fasting blood glucose, and postprandial blood glucose are instantly available

(2) Subsidizing the cost of patients’ travel to the hospital for visits

(3) Adding the patient’s network contact address, communicating and solving the patients’ problems at any time, requiring the patient to monitor blood glucose at home regularly and upload blood glucose data, continuing to conduct diabetes education

(4) Checking the number of medicine bags left by the patients at each return visit, as well as confirming the situation of medication

### Quality control and data collection

In order to maintain the high quality of the trial and ensure compliance with the protocol, all researchers and drug administrators participating in the study will receive rigorous training in accordance with the standardized operating procedure (SOP) manual. Researchers should accurately and clearly record patients’ information in accordance with the requirements of the CRF. The correction of error and its time will be marked. The recruited patients will be trained on how to collect stool samples canonically. The stool samples are collected from uncontaminated feces in the middle of the patient’s bowels. A sample collection box (made by Personalbio corporation) will be provided to the patients in advance. The patients will be informed that defecation should be done in the visiting morning as far as possible. The sampling volume is approximately 1/3–1/2 of the collection tube in the box and then to be separated into the 5-ml cryopreservation tubes by the researchers. Within half an hour, the samples will be transferred to the clinical biobank of Dongzhimen Hospital and stored in a refrigerator at −80 °C until testing. During the collection process, the samples are allowed to be temporarily stored at room temperature for no more than 4 h and temporarily stored in −20 °C refrigerator for no more than 24 h. All the data obtained from the research medical record will be uploaded to the digital database in the hospital. Eventually, all completed data will be uploaded to the Chinese Clinical Trial Management Public Platform (http://www.medresman.org.cn/).

### Data monitoring and auditing

The original data should be uploaded and transferred to the electronic case report form (e-CRF). Two administrators should perform 2 separate inputs and proofreading to ensure the accuracy of the data. The quality of the data will be managed through the Dongzhimen Hospital Data Monitoring Committee (DMC), where managers regularly check data sheets for omissions and errors to monitor the integrity of test data. They will also confirm that all associated documents like case report forms, medical charts, and initial hospital admission reports are up to date and monitor the audit trail of the digital database. From the first data entry, as well as each change, deletion, or increase, to the final entry, every digital footprint must be retained in the database system. The audit trail which includes change date, person, reason, and value should be consistent with the original data and open to the auditing of DMC. The DMC consists of endocrinology clinical experts, methodology experts, and biostatisticians, which are independent of the sponsor and have no conflict of interest with the research team. Original CRFs will be reserved at the research center in Dongzhimen hospital for 5 years for reference by researchers after completion of the study. Digital data will be accessed through the search of public title in the Clinical Trial Management Public Platform or the email communication with the first author.

### Statistical analysis

SPSS 20.0 software was used for the statistical analysis of data. Measurement data were described as mean (M) ± standard deviation (SD). Normality of continuous data was evaluated using the Kolmogorov–Smirnov test and Q–Q plot. If the paired samples were in accordance with normal distribution, the *t*-test of the paired samples was used for pairwise comparison. If not, the nonparametric test was adopted. If the two independent samples were in accordance with the normal distribution and homogeneity test of variances, the *t*-test of the independent sample was used. The *t*'-test was used when the variance was not in uniform and the nonparametric test was adopted when the samples were not in accordance with normal distribution. The covariance analysis will be performed regardless of the comparability in the baseline of Hba1c values of the two groups before treatment. Enumeration data like gender and medical history was described statistically by frequency. The chi-square test and Wilcoxon rank sum test were used to determine whether there was comparability between the two groups. All statistical tests are bilateral, and a *P* value ≤ 0.05 would be considered statistically significant.

## Discussion

Diabetes greatly impacts the quality of life and health of the patient, and the medical costs of its complications have become a heavy burden on individuals and the society. For a long time, the questions of how to effectively treat DN, reduce insulin resistance, and delay the progression of complications have always been the focus and difficulty of domestic and foreign research. TCM has the advantages of multiple targets, multiple pathways, and few side effects in the treatment of T2DM. With the further research on gut microbiota of T2DM, more and more attention has been paid to the specific effects of TCM on gut microbiota, but among which is lacking clinical studies. So it remains unclear how TCM treats diabetes by regulating gut microbiota.

Our preliminary research showed that the RG formula can effectively alleviate chronic inflammation and insulin resistance and improve reduce fasting and postprandial blood glucose in rats with T2DM [37]. Therefore, it is necessary to carry out the clinical research to explore the clinical efficacy and safety of drugs. In terms of its toxicology, the specific dose of Coptis root and ginseng was both certified as safe. In 2012, researchers concluded that the dosage of berberine for oral administration at 20.8 g/kg (or a berberine blood concentration of 0.168 μg/mL) was safe in mice, and the safety dosage for humans would be 2.97 g/kg of human body weight, which was much higher than the clinically recommended dosage of 15 mg berberine/kg of human body weight [38]. Ginseng had nearly no toxic effects in a regular intake (3–9 g) [39]. Only in the case of a high dose intake of ginseng (up to 15 g/day), there can be a small number of adverse reactions [40]. We expect the trial as safe and our findings may provide a new perspective to illustrate the mechanism of TCM treatment and promote the widespread application of it.

## Ethics and dissemination

The protocol has been approved by the Ethics Committee of Dongzhimen Hospital affiliated to Beijing University of Chinese Medicine (DZMEC-KY-2017-126). Any modifications to the protocol which may impact on the conduct of the study and potential benefit of the patient or may affect patient safety, including changes of study objectives, study design, patient population, sample sizes, study procedures, or significant administrative aspects, will require a formal amendment to the protocol. Such amendment will be agreed upon by the research group with the signature of the group manager and approved by the Ethics Committee of Dongzhimen Hospital prior to implementation and updated on the Clinical Trial Management Public Platform with regulations. The results of the clinical trial will be released to the participating physicians, referring physicians, patients, and the general medical community through peer-reviewed publications and conferences. Data that break the blind will not be presented prior to the release of mainline results. Recommendations as to the timing of presentation of such endpoint data and the meetings at which they might be presented will be given by the Steering Committee. This trial may terminate at the planned target of 6 months after the last participant has been randomized, or at an earlier or later date if the circumstances warrant. No later than 3 years after the collection of the 1-year postrandomization interviews, the completely deidentified data set will be delivered to an appropriate data archive for sharing purposes.

## Trial status

This study is under participant recruitment. The first patient was included on 12 Feb. 2018, and the recruitment will be finished before 28 August 2021. At present, 44 patients have been recruited.

## Supplementary Information

**Additional file 1:** The SPIRIT Checklist.

**Additional file 2:** The informed consent form.

## Abbreviations

T2DM: Type 2 diabetes mellitus
RG: Coptis root and ginseng
GCP: Good Clinical Practice
FBG: Fasting blood glucose
PBG: Postprandial blood glucose
FIL: Fasting insulin
C-P: Fasting C-peptide
IRI: Insulin resistance index
HOMA-IR: Homeostasis model assessment of insulin resistance
BMI: Body mass index
DMC: Data monitoring committee
GSDI: α-Glucosidase inhibitor
DPP-4: Dipeptidyl peptidase 4
GLP-1: Glucagon-like peptide-1
CRF: Case report form
IL-6: Interleukin-6
TNF-α: Tumor necrosis factor α
CRP: C-reactive protein
