# Supplementary material for: Effect of RG (Coptis root and ginseng) formula in patients with type 2 diabetes mellitus: a study protocol for a randomized controlled and double-blinding trial
Source: Trials. 2022 Apr 14;23:305. doi: 10.1186/s13063-022-06229-5 (PMC9009050; doi:10.1186/s13063-022-06229-5)
Supplement: Supplementary file 2 — Additional file 2: The informed consent form. [file 13063_2022_6229_MOESM2_ESM.doc]

**Informed Consent**

Dear patient,

Your doctor has confirmed that you have type 2 diabetes. We sincerely invite you to participate in the "Clinical Study on the Intervention of rhizoma coptidis and Ginseng on Type 2 Diabetes Based on the Observation of In gut Microbiota", which is conducted by Dongzhimen Hospital of Beijing University of Chinese Medicine.(No. 2018-JYB-JS9). Sixty volunteers are expected to participate in this study.In this experiment, you will be treated with "rhizoma coptidis and Ginseng" TCM granules for 12 weeks, during which free efficacy indexes and safety indexes will be checked.

I. There are strict inclusion and exclusion criteria for this study:

Inclusion criteria:

(1) Conformed to the 1999 WHO diabetes diagnostic criteria;

(2) Age 18-65;

(3) The target of diabetes control was achieved well, or after the induction period of less than 3 months, the target of diabetes control was achieved well, and the blood glucose was stable at fasting blood glucose < 7mmol/L and postprandial blood glucose < 10mmol/L;

(4) Informed consent, volunteers.The process of obtaining informed consent shall comply with GCP regulations.

Exclusion criteria:

(1) Age >=65 years old;

(2) Patients with serious heart, liver, kidney and other complications or with severe paroxysmal disease or mental illness;

(3) Pregnant or lactating women;

(4) Can not be combined with dietary control or do not use drugs according to the provisions and affect the efficacy;

(5) Inflammatory bowel disease;

(6) Recent use of antibiotics;

(7) Those who refuse to participate in the study or cannot sign the informed consent.

This study plan has been approved by the Ethics Committee of Dongzhimen Hospital of Beijing University of Chinese Medicine and approved for clinical study.

2.the possible benefits of research

If you do participate in this study, you may benefit from this study, such benefits include:

The free drugs and tests provided in this study are as follows:

(1) If you meet the drug intervention criteria and do not meet the exclusion criteria, this study will provide you with free lifestyle guidance for diabetic patients, free 12-week study drugs or simulants, and free inflammatory indicators and intestinal microbiota testing.The traditional Chinese medicine in this subject has been verified by previous studies and has a clear role in reducing postprandial blood glucose and improving insulin resistance.

(2) If you do not meet the drug intervention criteria or meet the exclusion criteria, this study will still provide you with free lifestyle guidance for diabetic patients, and provide free tests for inflammatory indicators and intestinal flora.

(3) Self-paid items during the research period: blood glucose monitoring three times a week, five times a day, blood glucose test paper: 59 yuan/box, 3 boxes in total 177 yuan.

3.Possible risks in the process of research

So far, no obvious adverse reactions have been found in the existing clinical studies.However, in the course of the study, abnormal damage or unexpected adverse reactions cannot be ruled out.Case reports show that there is a certain risk of constipation after taking Coptis chinensis, but it can be gradually relieved after adjusting the dietary structure.You need to visit the hospital regularly during the study period, which may cause inconvenience to you.If you participate in this study, if you have adverse reactions or adverse events related to this study during the study period, such as severe gastrointestinal reactions and allergic reactions, please contact the investigator for help, and the investigator will give free examinations and relevant medication treatment.

Agreement Signature Page

Project title: Clinical Study on the Intervention of rhizoma coptidis and Ginseng on Type 2 Diabetes Based on the Observation of In gut Microbiota.

Research Unit: Dongzhimen Hospital, Beijing University of Chinese Medicine.

I have read the above introduction to the study and have had the opportunity to discuss and ask questions about the study with doctors.All my questions related to the research were answered satisfactorily.

I am aware of the possible risks and benefits of participating in this study.My participation in the study is voluntary and I am sure that I have had sufficient time to consider it and understand that:

I can always ask my doctor for more information about the study.

I can withdraw from the study at any time without discrimination or retaliation.

I also know that if I withdraw from the study, especially if I withdraw due to medication, it would be beneficial for me to inform my doctor of any changes in my condition and make a final evaluation.

If I need to take any other medications due to a change in my condition, I will consult my research physician first.

I agree with the Ethics Committee of Dongzhimen Hospital of Beijing University of Chinese Medicine to have access to my research materials.

I will obtain a signed and dated copy of the informed consent.

In the end, I decided to agree to participate in the study and promised to try to follow the doctor's advice.

Patient Signature:

Date:

Phone number (mobile phone) :

I confirm that I have explained to the patient the details of this study, including the rights and possible benefits and risks, and have given him a signed copy of the informed consent.

Signature of doctor:

Date:

Contact number (mobile) :

**受试者知情同意书**

尊敬的患者：

医生已经确诊您患有2型糖尿病。我们诚挚地邀请您参加这项由北京中医药大学东直门医院负责的 “基于肠道菌群观察黄连人参“药对”干预2型糖尿病的临床研究”项目。任务书编号：2018-JYB-JS9。本研究预计将有60名受试者自愿参加。在本次实验中，您将进行为期12周的“人参、黄连”中药颗粒剂治疗，期间会提供免费的疗效指标、安全性指标检查。

一、本研究有严格的纳入标准及排除标准：

纳入病例标准：

(1) 符合1999年WHO的糖尿病诊断标准；

(2) 年龄18-65岁；

(3) 糖尿病控制目标达较好，或经过3个月以内的导入期，糖尿病控制目标达较好者，血糖稳定在空腹血糖＜7mmol/L，餐后血糖＜10mmol/L；

(4) 知情同意，志愿受试。获得知情同意书过程应符合GCP规定。

排除病例标准 ：

(1) 年龄>=65岁；

(2) 有严重心、肝、肾等并发症或合并有严重发作性疾病、精神病患者；

(3) 妊娠或哺乳期妇女；

(4) 不能配合饮食控制或不按规定用药而影响疗效者；

(5) 炎症性肠病；

(6) 近期应用抗生素者；

(7) 拒绝参加本研究或不能签署知情同意者。

本研究方案已经通过北京中医药大学东直门医院伦理委员会审核，同意进行临床研究。

二、研究的可能的获益

如果您确定参加本研究，您将可能从本项研究中受益，这种收益包括：

本研究提供的免费药物及检测项目如下：

1.如果符合药物干预标准且不符合排除标准，本研究将为您免费提供糖尿病患者生活方式指导，免费提供12周的研究药物或模拟剂，并免费提供炎性指标及肠道菌群检测。本课题中药经前期研究验证，具有明确的降低餐后血糖、改善胰岛素抵抗的作用。

2.如果不符合药物干预标准或符合排除标准，本研究仍将为您免费提供糖尿病患者生活方式指导，并提供提供炎性指标及肠道菌群的免费检测。

3.课题期间自费项目：一周三次，每日五次血糖监测，血糖试纸：59元/盒，3盒共177元。

四、研究过程中可能的风险

到目前为止，已有的临床研究未发现研究用药物有明显的不良反应。但在研究过程中，不排除发生非正常损害或非预期的不良反应。个案报道显示服用黄连后存在一定的便秘风险，但经调整膳食结构后可逐渐缓解。您在研究期间需要按时到医院随访，这些可能会给您带来不便。如果您参加本研究，在研究期间，如出现与本研究有关的不良反应及不良事件，如严重的胃肠道反应及过敏反应，请与研究者联系以获得帮助，研究者将给予免费的检查及相关药物处理。

**知情同意书**

**同意签字页**

课题名称：基于肠道菌群观察黄连人参“药对”干预2型糖尿病的临床研究

研究单位：北京中医药大学东直门医院

我已经阅读了上述有关本研究的介绍，而且有机会就此项研究与医生讨论并提出问题。我提出的与研究有关的问题都得到了满意的答复。

我知道参加本项研究可能产生的风险和受益。我参加研究是自愿的，我确认已有充足时间对此进行考虑，而且明白：

我可以随时向医生咨询与研究有关的更多信息。

我可以随时退出本研究，而不会受到歧视和报复。

我同样清楚，如果我中途退出研究，特别是由于药物的原因退出研究的，我若将我的病情变化告诉医生，并进行最后的评价，这将对整个研究十分有利。

如果因病情变化我需要采取任何其它的药物治疗，我会事先征求研究医生的意见。

我同意北京中医药大学东直门医院伦理委员会查阅我的研究资料。

我将获得一份经过签名并注明日期的知情同意书副本。

最后，我决定同意参加本研究并保证尽量遵从医嘱。

患者签字： 日期：

联系电话（手机）：

我确认已向患者解释了本研究的详细情况，包括权利以及可能的收益和风险，并给其一份签署过的知情同意书副本。

医生签字： 日期：

联系电话（手机）：_ _
